# How do drug users define their progress in harm reduction programs? Qualitative research to develop user-generated outcomes

**DOI:** 10.1186/1477-7517-1-8

**Published:** 2004-08-26

**Authors:** Terry Ruefli, Susan J Rogers

**Affiliations:** 1 New York Harm Reduction Educators, Inc (NYHRE), 903 Dawson St., Bronx, New York 10459; 2 Academy for Educational Development (AED), 100 Fifth Ave., New York, New York 10011

## Abstract

**Background:**

Harm reduction is a relatively new and controversial model for treating drug users, with little formal research on its operation and effectiveness. In order to advance the study of harm reduction programs and our understanding of how drug users define their progress, qualitative research was conducted to develop outcomes of harm reduction programming that are culturally relevant, incremental, (i.e., capable of measuring change), and hierarchical (i.e., capable of showing how clients improve over time).

**Methods:**

The study used nominal group technique (NGT) to develop the outcomes (phase 1) and focus group interviews to help validate the findings (phase 2). Study participants were recruited from a large harm-reduction program in New York City and involved approximately 120 clients in 10 groups in phase 1 and 120 clients in 10 focus groups in phase 2.

**Results:**

Outcomes of 10 life areas important to drug users were developed that included between 10 to 15 incremental measures per outcome. The outcomes included ways of 1) making money; 2) getting something good to eat; 3) being housed/homeless; 4) relating to families; 5) getting needed programs/benefits/services; 6) handling health problems; 7) handling negative emotions; 8) handling legal problems; 9) improving oneself; and 10) handling drug-use problems. Findings also provided insights into drug users' lives and values, as well as a window into understanding how this population envisions a better quality of life. Results challenged traditional ways of measuring drug users based solely on quantity used and frequency of use. They suggest that more appropriate measures are based on the extent to which drug users organize their lives around drug use and how much drug use is integrated into their lives and negatively impacts other aspects of their lives.

**Conclusions:**

Harm reduction and other programs serving active drug users and other marginalized people should not rely on institutionalized, provider-defined solutions to problems in living faced by their clients.

## Background

Harm reduction programs operate with the assumption that some people who engage in high-risk behaviors are unwilling or unable to abstain. Using a "low-threshold approach," they do not require that clients abstain from drug use in order to gain access to services, nor expect adherence to one service to be eligible for another. Rather than having abstinence goals set for them, clients in such programs take part in a goal-setting process, an approach that has been shown to correlate consistently with retention and success [1-3]. Providers help clients make connections among their complex attitudes, behaviors, and the change they are trying to pursue as a result of an interactive process – not a dogmatic format. Behavior change is regarded as incremental and based on the premise that people are more likely to initiate and maintain behavior changes if they have the power both to shape behavioral goals and enact them.

Research on harm-reduction/syringe-exchange programs has been limited largely to demonstrating their success with reducing the transmission of HIV/AIDS among drug users [4-11]. While this is an important accomplishment, little is known about their impact in assisting drug users in making changes in life conditions, circumstances, and quality of life. This is partially because few efforts have been made to establish appropriate measures of client and program progress in these areas.

The traditional field of drug treatment has generated many assessment tools including the Addiction Severity Index used extensively for treatment planning and outcome evaluation [12]. This tool, and others like it, such as the Chemical Dependency Assessment Profile (CDAP) [13] and the assessment forms created at the Institute of Behavioral Research at Texas Christian University (TCU/DATAR), generate severity ratings that are subjective ratings of the client's need for treatment derived by the clinician. The ASI interview asks questions related to domains or "problem areas" in substance abusing patients that have been determined by clinicians, not the patients themselves. Thus, despite the formally established validity and reliability of the tool, and others like it, the measures are developed from the perspective of the clinician and researcher and are designed to generate information that is consistent with their view of the world, not the world of drug users. Given the tenets of harm reduction in which drug users participate in their own goal setting, such tools lack cultural sensitivity and relevance. Denning's work on harm reduction psychotherapy (2000) is considerably more grounded in the life circumstances of drug users. Her Multidisciplinary Assessment Profile (MAP), a baseline assessment tool to use with chemical dependency clients, however, was not designed to generate information about what drug users consider to be realistic goals and progress towards these goals. If service providers are to guide an effective interactive process of goal setting, it is important that they understand the parameters of realistic, incremental behavior change from the perspective of the client.

Since the development of the ASI, there has been growing movement to acknowledge the value of participatory research in which the "subjects" of research become directly involved with shaping the research agenda and designing data collection tools. Such an approach empowers the community participating in the research so that members are not objects acted upon but rather partners in an endeavor to improve their circumstances. This approach increases the cultural appropriateness of the way the research is conducted, the potential validity and reliability of the data that are generated, and the utility of the results.

In order to advance the study of harm reduction programs and our understanding of how drug users define their own progress, we conducted participatory research to develop outcomes of harm reduction programming. The goal of the research was to involve program clients in a process that would generate valid measures that are 1) culturally relevant to the way they see the world and live their lives; 2) incremental – i.e., capable of measuring small changes, and 3) hierarchical – i.e., capable of showing how clients improve over time. This article summarizes information on the research methods used and the outcomes that were generated.

## Methods

The research study was conducted in two phases. In the first phase, drug users participated in a group process using nominal group technique (NGT) [15] to develop the outcome measures. In the second phase, other drug users participated in focus group interviews to reflect on the measures developed and their validity for the drug-using population. Below, the sample and methods of the two phases are described in more detail.

### Sample

Study participants were recruited from the New York Harm Reduction Educators, Inc. program, which has delivered comprehensive services to over 40,000 enrollees at six sites located in East Harlem and the Bronx, New York. To involve recipients in the study, clients were recruited by program staff and given a $10 incentive for participation. The study was advertised at the main program site, and a stratified convenience sample of approximately 120 clients was recruited for phase one of the study, and 120 for phase two, with some duplicate count of clients who participated in both phases of research. The sample was stratified by neighborhood, by time in the harm-reduction program, and by whether participants took part in the syringe exchange only or accessed a fuller range of services. The demographics of the sample closely represented the larger program and included 26 % African American, 50% Latino, and 24% white; 72% male and 28% female; 17% ≤ 29 years of age; 36% between 30–39, and 46% ≥ 40 years of age.

### Methodology

In the first phase of the research, a facilitator independent of the program used NGT with 10 groups of approximately 12 individuals per group. This group process provides a structure for small group meetings so that client participation is maximized and judgments effectively pooled. The technique was especially helpful in establishing priorities in that it neutralized differences in status and verbal dominance among participants. Before the present study, NGT was used successfully with clients in the program to identify areas of life functioning that people like themselves (i.e., drug users) deemed to be the most important and meaningful in their lives. The top 10 "life" areas were money (income); housing; food (nutrition); family relations; self-improvement; connectedness to services/benefits/programs; dealing with negative feelings (mental health); health problems (physical health); and legal and drug use problems.

To generate items in a scale for every outcome listed above, 12 people were recruited for a group that used several NGT process steps. First, the facilitator asked the group to contemplate a question related to the outcome of interest. For example, "What ways do people in your circumstance make money?" was asked to generate the outcome of source of income; "What ways or places do people in your circumstance get something good to eat?" was asked to generate the outcome of source of nutrition; and "What are the types of places that people in your circumstance live?" was asked to generate the outcome of housing. Next, the members of the group brainstormed their answer(s) to the question posed to them and the facilitator recorded these answers on a large chart. The facilitator continued to call upon the members until all ideas were recorded.

Next, all group members received a packet of 10 index cards numbered 1 to 10. The facilitator engaged the group in ranking the ideas according to every individual's order of preference. This step started with the facilitator asking individuals to take out the index card marked #1. She then asked every group member to select from the list that idea that he/she considered the best (i.e., the best way to make money, the best way/place to get something good to eat, the best place to live, etc.) and write it down on card #1. She then asked everyone to take out card #10 and select the idea that he/she considered the worst (i.e., the worst way to make money, the worst way/place to get something good to eat, the worst place to live, etc.) and write it down on card #10. Finally, she led them through a similar ranking process with cards #2 – #5, used by the individuals to record their "next best" preferences and cards #6 – #9 to record their "next worst" preferences.

This group work resulted in 10 scaled outcomes. In the second part of the research, 10 focus group interviews were conducted to allow more of the target population to reflect on the validity of the measures and further explore the meanings of the scaled items based on the lives of drug users. In most cases, a completely different group of drug users who had participated in the NGT process for a certain outcome were selected to participate in the focus group related to that same outcome.

### Data Analysis

The analysis of the data from each NGT group for every outcome was done by first eliminating all ideas that received no votes. The remaining data were then analyzed by: 1) determining the total score for all remaining ideas (the individual score of each idea was based on the card number on which it was recorded – i.e., 1–10 – and the total was the sum of individual scores); 2) determining the mean score for every idea (dividing the total score for an idea by the total number of votes – i.e., cards received for the idea; 3) rank-ordering the ideas by mean score; and 4) grouping the 40 or more individual ideas with similar mean scores by larger concepts so that every outcome had about 12 hierarchical, scaled items from best to worst, with mean scores from low to high.

## Results

The results of the preliminary research are displayed in Table 1 (see Additional file: 1) showing every individual outcome and the hierarchical scale of items that measure incremental change from better to worse. "Better" items in every outcome, near the top of the scale, were those items that had received a mean score of 5 or better while "worse" items, near the bottom of the scale, were those items that received a mean score of 6 or higher. A summary of results for every outcome is presented below.

### Money (income)

The outcome 'money (income)' includes a hierarchical scale of 11 items measuring better to worse 'ways of making money'. According to the clients, better ways of making money were from entitlements; a legitimate job; from family (i.e., through marriage, inheritance); or borrowing from others. Worse ways of making money were from hustling (i.e., conning or informing to police); stealing (i.e.,"boosting" – stealing); drug trade (e.g., selling, holding, transporting); panhandling; more serious criminal activity (i.e., credit card fraud, robbery, hitman); sex work; and selling blood or body parts.

When other program clients reviewed this outcome scale, they felt it reflected their lives overall. One exception was that many felt that "job" (employment, peddling, odd jobs, volunteer) should be at the top of the scale rather than "entitlements." Some saw entitlements as "an easy way to make money – "it's a way of survival," – while having a job offered more independence and feelings of self-esteem. Other changes suggested by individuals were that "stealing," "drug trade" and "hustling, police informant" should be further down on the scale while "panhandling" should be further up. Strong negative feelings were expressed about being a police informant;" "Where I come from, when out on the street..., you don't inform the police! Police will lie to you and use you, then throw you back to the dogs and you're dead."

Focus group participants spoke about what money meant to them and what life was like when one made money via drug trade, sex work or selling blood or body parts. Like most people, they felt having money offered a sense of freedom and independence. In addition, many felt that having it contributed to self-esteem and allowed you to help others you cared for: "It lets you help a family member who needs help"; "It helps support my spouse... and give some money to my son when I can." Regarding money making from drug trade, study participants felt, overall, that it was unstable and risky.

It's like addiction. It's an adrenaline pump ... that keeps you in it for so long. You do it to keep your habit going... If you sell what you use, it's not good. You wind up doing all the drugs, then you have to run for your life from the dealer.

It's an unstable life...and the consequences are great if you get caught. You go to jail, get clean, come back out, and start all over again. It's a never-ending cycle.... Eventually your luck runs out and you either go to jail or get killed.

Sex work was also described in negative terms:

That's the last thing you want to have to do, man or woman. Selling your body is the worst thing you could do for yourself. You can get HIV, hepatitis C, or even get killed. You don't know who this person is that you are engaging in this sex at with. He could be a serial killer.

Selling blood and body parts were described as both limited and, again, risky:

Now you are put on a computer and once they have a record of your blood test, you can't do it because they can screen you from sight to sight... If you sell a body part, they might not wait for you to die to get it, they might hunt you down and kill you.

### Housing

The outcome 'housing' is made up of a hierarchical scale of 11 items measuring the better to worse places to live. Based on client input, better places to live were a house or apartment that you rented or owned; a friend's home; a drug program; a family member's home; housing provided through a social program; institutionalized housing, such as a shelter or hospital; and homelessness that was considered "least severe (e.g., sleeping on the subways or at a bus station). Clients considered worse places to live to be jail, homelessness considered to represent an intermediate level of severity (sleeping in cars, bathrooms, hallways, abandoned buildings) or the most severe level (in tunnels, caves, sewers, parks, on a roof).

When other program clients reviewed this outcome scale they agreed, overall, that it reflected the reality of their lives. A few suggested minor changes in the scale could be made such as moving "jail" as a housing option to the very end of the scale because, as one said, "I don't want my freedom taken away – it's degrading and lowers your self-esteem being subject to a strip search at any time." A few others suggested putting "apt/room that you rent or own" before "friend's home." Most felt that it was better to have your own place than to live with family or friends because "it makes you feel independent, feel human – it's an accomplishment." They also offered some insight on what it was like being a drug user and living with a family member vs. a friend.

A family member would let you get away with more than a friend would. With a friend, you would have to be on time, with all of your part of the rent money, food money, and clean up after yourself. With family you might dib and dab a little with the rent money or get out of doing some things around the house. However, when you're on drugs, going to your family is not good because they can give you the boot.

Other "windows" of insight into how drug users deal with housing came in participants' discussion of being in a drug program, institutionalized housing (shelters, hotels, hospitals, SROs), and living on the street. Participants thought that drug programs offered an important option for housing but also saw them as a last resort:

They give you structure and are stable – you can get food, clothes and confidence. Last resort when you have no place to stay and have no money... It's the place to go if you want to change your life around.

Some program clients saw institutionalized housing as a crutch, while others discussed the advantages and disadvantages of various types of institutionalized housing:

I became "shelterized" after being in a shelter for several years. I got locked into a routine where you don't want to take care of yourself because the basics are provided for you. Life was sweet, too sweet – I had no responsibility.

Some hotels, depending on where they are located, have a high level of theft with no security.

Hospitals will take you in depending on the weather and your state of mind. The homeless quarters and the psyc ward are often connected. Sometimes you have to fake it, like you need psychiatric care, if you want to get off the street for a while. If you act like you are going to harm yourself, they give you a bed fast.

SROs are like apartments but they have rules... Some don't allow company but others do. You have to sign visitors in and out at the desk.

Tier 1 and 2 housing is for families. But it's only temporary – 30 to 60 days. Then you have to pack up everything and move somewhere else.

Section 8 housing has a lot of limitations. You have to be a family that is homeless living in a shelter, a victim of domestic violence, or in the witness-protection program. You can't have any felonies on your record. Some of section 8 is only available if you are HIV positive.

Generally, clients felt that living on the street was a last resort but an option that could work:

If you bum everything out by not following rules, stealing, looking for fights or taking drugs, then the street will become your home... But you can make the street work for you if you know how to survive. I did. You pick your area and take a claim for it. I had a half car that was my roof... I even evicted some people from my neighborhood because they didn't act right. We had our own rules.

### Food (nutrition)

The outcome 'food (nutrition)' is made up a hierarchical scale of 11 items measuring the better to worse 'ways/places to get something good to eat'. According to the clients, better ways/places to get something good to eat were to cook food yourself; from friends or family; from a supermarket; from place that gave out free food (from soup kitchen, shelter, pantry, social gathering); buying food (with food stamps or from money earned); or from a restaurant. Clients considered worse ways/places of getting food were from begging or stealing; from institutions (hospital, jail), from trying to provide for yourself (hunting, fishing); and, lastly, from the garbage.

After other program clients reviewed the developed scale, they generally agreed that it reflected their lives. The exceptions were that some felt that "buying food" should be placed higher in the scale, preferably at the top. The rationale was that before you could cook food yourself, you needed to buy it. In addition, others felt that "food from facilities" and "providing your own food" should be placed before stealing food in that stealing food involved risk and possible repercussions.

Study participants spoke about what they considered "something good to eat." They most often mentioned that food needed to be tasteful, although not necessarily nutritious. The feelings that they associated with getting something good to eat were "feeling good," "wanting to act civil," and "wanting to treat people better." Feelings associated with not getting something good to eat were "feeling cheated" and "developing an instant attitude." They also discussed why "cooking food yourself" was higher on the scale than "going to a restaurant" or having others prepare the food for you. The importance of self-sufficiency emerged when participants spoke about the value of "cooking it the way you want it" and the feeling of comfort that came from "doing it yourself" and being able to "be at home with my girl and be able to afford a full-course meal."

Participants had much to say about the topic of "free food" and the better/worse places of getting. Although "the price was right," and they were all aware of "street sheets" listing several places to go for free food, they also spoke about traveling long distances, waiting on long lines, walking up several flights of stairs, and having to have a referral and register with a program to get food. The factors that affected their decision about where to get food were the attitudes of the staff, the quality of food offered (i.e., brand names were preferred over generic, U.S.D.A. – grade foods), and whether the program also offered other needed services (e.g., some pantries offered services like showers and laundry facilities). Some participants contended that some program staff in pantries "pick through the groceries and bag up the best stuff for themselves and friends."

Other revealing insights that drug users had about food were that they did not feel it was ever necessary to steal or beg for food: "There are plenty of places to get food. Anyone you see stealing food or begging is doing it for a profit, to be able to purchase something else." A number of participants referred to "dumpster divers" (people who eat street or building garbage) as people who were mentally ill and took great risk of eating contaminated food. Most felt more comfortable eating leftover, pre-wrapped food from fast-food restaurants than resorting to street dumpsters.

### Family relations

The outcome family relations includes a hierarchical scale of 15 items measuring the better to worse 'types of family relations'. At the top of the scale clients considered better ways of relating to family to include loving your family; taking part in special family gatherings; having positive communication (open, honest, tolerant); interacting directly with members (playing games, picnics, talking about family history); arguing; showing support and respect, spending high quality time; and engaging in passive contact (movies, TV, reading the Bible). Clients felt the worse ways of relating to family were showing a lack of respect between members (being stigmatized/disrespected for who you are, talking about drugs around children); members' having negative attitudes toward one another (envy, judgment, alienation); conflicting lifestyles; engaging in abusive relations (incest, sexual abuse, violence); having difficulties with financial support; abandoning a family member; or being deceitful (stealing, gossiping, lying).

Upon reflecting on the scaled outcome later, other program clients generally agreed that the scale reflected their lives, with a few exceptions. Several felt that "arguing," in the middle of the scale, should be placed further down on the scale as they saw it as a way of relating that often leads to abuse. In addition, there was some disagreement on the order of the items considered the worse types of family relations. Some clients felt that "abusive relations" should be listed last. These individuals spoke painfully about how abusive relations in their childhood had damaged them throughout life, and others spoke about how exposure of the abuse within their family had created lasting division between members.

Client input on the items "love," "respect," and "negative attitudes" illuminates the meaning of these terms in the lives of drug users. "Love" was seen as a building block and foundation for family relations. It was equated with respect among family members, with one client asserting that "Love for my family may mean not spending time with them so that I do not expose them to my drug use." Clients felt that indicators of family respect were listening, letting people have their say, giving people the benefit of the doubt, and living life your way without interfering with others. Clients reflected on the negative attitudes they had experienced around family members. Along with outwardly judgmental remarks, clients also experienced a great deal of nonverbal behavior that they interpreted as negative attitudes. Examples included when they walked into the room where family members were conversing, and people suddenly stopped speaking or hid their purses. Overall, clients felt that a family member's drug use should not necessarily engender negative attitudes among other family members and that their families needed to learn more about understanding the harm reduction approach.

### Connection to services, programs and benefits

The outcome 'connection to services, programs and benefits' consists of a hierarchical scale of 12 items measuring the better to worse 'types of services/ programs/benefits available to drug users'. Clients felt better types of services to connect with were those related to housing; HIV/AIDS assistance; mental health; drug treatment; entitlements (i.e., public assistance, SSI, and social services); and harm reduction (outreach, needle exchange, condoms). They considered the less preferred (i.e., worse) available services to be those in mainstream institutions (churches, library, legal services); "getting-connected" services (escort services, resource directories); support services (12 step, women's groups); family-prevention services (parenting skills, domestic violence services); stress reduction (acupuncture, field trips); and work (WEP) programs.

Later, when other clients reviewed the developed outcome, most felt that it overall reflected their lives. The one change that a sizable number of clients called for was to put harm reduction services farther up on the scale. The value they placed on this type of service was shown in a number of comments:

Harm reduction has taught us a lot about taking care of yourself physically, mentally and emotionally. If you are using drugs, it teaches you how to use drugs safely and in a safe environment. If you want to stop using, there are places to go to get the help you need. If you are out on the street hustling, selling your body it teaches you about using protection.

Harm reduction is very important because it taught me a lot about how to take care of myself, manage my drug use, use my needles properly, and reduce my stress.

In addition, certain individuals, based on their circumstances, made other suggested changes. One client who disclosed himself as HIV sero-positive said that "AIDS-related services" was the best service on the list for him. Another client remarked that "drug treatment" would need to be listed before housing since you are required to be drug-free to get housing. Still another felt that all the listed services were important "because they can assist me in preparing for my future."

The clients discussed why "support services" (12-step programs, advocacy groups) were fairly far down on the scale. Overall, they felt this was because participation in these programs was dependent on giving up drugs, which some people are not ready to do. They also felt some people do not agree with the philosophy of the programs nor are they ready to be in a group environment.

Clients also spoke at length about why the "WEP (Work Related) program" was listed last on the scale. Although they thought it might benefit people who have no skills, they felt, overall, that the program was degrading.

For those who may have skills, it's kind of degrading in a way because you are working for a check that you are receiving from public assistance.

For a single person you might get $68.00 every 2 weeks and you are doing the same kind of work that the people earning above minimum wage are getting.

You can be working in the Parks department, cleaning people's toilets or picking up paper in the street for the sanitation dept. Some of it can be real degrading and discouraging.

### Self-improvement

The outcome 'self-improvement' consists of a hierarchical scale of 12 items measuring the better to worse 'ways of improving yourself'. Study participants felt that better ways of improving yourself were having a better relationship with yourself (self-love, respect) and with others; getting and staying clean from drugs; being spiritual; taking part in self-help groups (12-step programs, support groups); working or developing work skills; and engaging in stress-reduction activities. They considered less preferred or "worse" ways of improving yourself to be helping others; taking care of yourself (i.e., going to dentist, taking medications, dieting, going to gym); being more responsible (i.e., living on a budget, accomplishing goals); behaving yourself (stop lying, stay out of trouble); and having a hobby (i.e., art work, boating, fishing, hunting).

After a different group of program clients had reviewed this outcome, most felt that it adequately reflected their lives. A few individuals suggested that "caring for self" and "being more responsible" (i.e., items #9 and #10) should be listed further up on the scale. In addition, one individual felt that "becoming more spiritual" should be first on the scale, "because if you have God in your life, everything else will fall into place."

Program clients were asked about the meaning of "self-improvement," "self-respect," "relating to others" and other items as they appeared on the scale. Concerning self-improvement, clients often thought of the topic as one that involved personal goal attainment.

Setting goals that are positive and reaching them. Then setting another and reaching it, one step at a time.... Setting up a network that will help me to build a foundation of positive aspects in my life that I can follow.

The clients described self-respect as requiring self-esteem, as being linked with showing respect for others and with how you physically appear to others, and as dependent on managing your drug use.

If you have self-esteem and care for yourself..., respect will come.

By you respecting yourself and wanting to be treated a certain way, you know you have to respect others to get it back in return.

If I looked better, I would feel better about myself.

A lot of time when people are drugging, they get caught up in a lot of things and before they know it, they have done some things that have cost them their self-respect, so getting it back is important to be able to get on with your life.

In clients' discussion of the meaning of "relating better to others," several indicators emerged such as honest communication; holding an intelligent conversation about yourself; being comfortable relating your feeling to others; and listening. Their thoughts on "getting/staying clean" (item #3) demonstrated the challenges they face and the degree to which their lives must change to stay clean.

It was a hard process for me because I would always fool myself that it wasn't the right time.... You can't do it for someone else, it has to be for you.

It took me becoming homeless to decide that I had to make some changes in my life. Now that I have a new apartment, I want to keep it. My budget won't allow me to get high and keep my rent paid.

Once I got out (of jail), my body was clean but my mind was still dirty. Mentally I still wanted to do drugs... I had to leave people, places and things alone because I feel powerless over the influence of others. Being around positive people and getting the support of groups helped me stay clean.

Clients also provided rich detail on what they meant by "behaving myself" (item #11), including this response:

It's the whole package. Your attitude, the way you talk, the language you use. When you start to change your life for the better, everything changes. You don't use a lot of 4-letter words. You want to socialize with different people in a different atmosphere. Not getting high where you work at; being more responsible.

Finally, the clients were asked why "working/developing work skills" (item #6) was as far down on the scale as it was. Most acknowledged that this was a goal that many drug users are not yet ready to achieve, given their difficulty functioning in an environment that they are not used to.

If you are coming into work and you are in this other world where you are not sick (to others), but you are not well either, it is hard to function. You have to have a functional mind that is able to concentrate on work...and for a lot of people, they are not there yet.

Alternatively, their discussion suggested that volunteering was a better way to approach the world of work: "I started volunteering here at NYHRE, and I intend to go to computer school so I can get a better job."

### Mental health

The outcome 'mental health' consists of a hierarchical scale of 13 items measuring the better to worse 'ways of handling negative feelings'. Study participants felt that better ways of handling negative feelings were getting informal support; (from friends, support groups), spiritual help (going to church, praying); or professional help (from a doctor, counselor); working; engaging in diversions (interacting with children; going to ball game or the beach, singing), or in stress reduction (smoking, massage, sex) and physical activities (exercise, cooking, sports). They considered worse ways of dealing with negative feelings to be engaging in violence against self (suicide, bulimia, anorexia); outward violence (hurting others, breaking things); bringing negative feelings into social relations (into marriage, when visiting someone in jail); withdrawing ("isolating"); and engaging in illicit activity (working the streets, using drugs, gambling).

When another group of program clients reviewed the scaled outcome, they saw the relevance of all the items and agreed on the general order of the items in the scale. A few clients suggested some minor adjustments in the scale, however. For example, a few felt that "professional help" (item #3) should be considered the best way of handling negative feelings, rather than "get support" (from friends, support groups). For the most part, however, the majority of the clients agreed that getting support from friends and support groups was more functional for people in their circumstance than going to a professional because of issues of availability. As one person put it, "The drug man never sleeps" and people involved in this culture need easy access to those who can help them with their negative feelings.

When you are out in the street drinking and drugging, there is something going on at every corner 24 hours of the day. Support from friends and groups are available to you on those off hours when "professional help" is not.

This was also the rationale for few clients as to why "spiritual help" should be placed before professional help – you can pray at any time. Other individual clients felt that "social relationships" should be further up on the scale because peers and loved ones were often the most understanding.

You need communication with someone that understands you and is willing to put up with your shortcomings. Problems do arise if one gets high and the other does not, but you can usually work this out.

Things get tough sometimes but she helps to keep the balance in the relationship. My spouse helped me with my addiction.

In regard to how drug users resorted to abuse in deal with negative feelings, the clients often referred to circumstances involving drugs. When verbal abuse did not work, they often resorted to physical abuse.

A spouse will be abused when you want your drugs and you don't have the money. You know that she has the money but she won't give it to you.

When selling drugs and someone comes to you with short money, even if it is only $1, he might get his butt whipped.

### Dealing with health problems

The outcome 'dealing with health problems' consists of a hierarchical scale of 12 items measuring the better to worse 'ways of handling health problems'. The clients in the study considered better ways of handling health problems to be using home remedies (external and internal cleansing, praying); stress reduction; drug treatment/therapy; "clean living" (i.e., reduced drug use, taking meds, stopping smoking); seeing the doctor; and getting health screenings. They felt less preferred (i.e., worse) ways of dealing with health problems were maintaining a good diet, getting health education information, exercising, using alternative therapies (i.e., fasting, herbs, psychic readings, witch doctors), exhibiting negative emotions (depression, denial, suicide, anger); and using illicit drugs.

After another group of program clients reviewed this outcome scale, they generally felt that it reflected their lives, with a few exceptions. Several felt that "see a doctor," "educate yourself" and "alternative therapies" should be higher on the scale. Most clients felt that "home remedies" should stay at the top of the scale because "they work the best." When they spoke about their experiences with doctors, it often was not positive.

The waiting is horrible. As an inpatient, you could die before you see a doctor. Once you are identified as an addict, whether on methadone or still using drugs, you're discriminated against.

Sometimes I am too leery to go to see a doctor. I may wait for someone else to go to the doctor first and then get their opinion.

When asked about the health problems they encountered, client usually mentioned serious conditions (cancer, STDs, HIV, pneumonia), indicating that ailments were not a health program unless they had become serious.

Regarding drug treatment, clients saw it as a positive way to deal with health problems, with certain parameters. "Drug treatment is not going to help you if you are not ready to stop using... It won't help you unless you have a follow-up plan like a support network at a church, family or groups, and being around positive people." Clients also discussed how other items on the scale were related to their drug use. Several felt that "using illegal drugs" should be at the very bottom of the scale, but opinions about this varied based on level of drug use. Clients knew that drugs could eventually bring about bad health but were often so out of touch with their feelings while doing drugs that they thought they were healthy:

When I was on a constant run (doing drugs), I didn't get sick. Thought I had a wonder drug. Didn't feel anything; drugs preserved me. I didn't get headaches, toothaches or colds. If I was sick, the drugs controlled my inner body, I couldn't feel a thing.

They felt that the item on the scale "educate yourself about health" was especially important for drug users who are often controlled by their substance:

No one used to take vitamins because your drug controlled your mind. You couldn't eat properly because you had to get high first. Education about my health has helped me make some changes. Before I didn't go to a doctor. Now I make an effort to go on a regular basis.

### Dealing with drug use problems

The outcome 'dealing with drug-use problems' consists of a hierarchical scale of 17 items measuring the better to worse 'ways of handling drug-use problems'. Study participants felt that better ways to handle drug-use problems were to admit the problem (and make amends with family); engage in religious activity (go to church, pray); get social support (from support groups, asking for help, making new positive friends); go into drug treatment; quit using drugs; get professional help (therapy, education about drugs, medications); stay distracted (keep busy, play with kids); and avoid the drug culture (avoid places that trigger drug use, drug paraphernalia). Clients considered the less helpful (i.e., worse) ways of handling drug-use problems were to follow a treatment plan (go to the hospital, take and not sell medications), get family support or spiritual guidance (from 12-step programs, minister); be in jail; be honest with yourself (reflect on past behaviors and pain associated with use); be deceitful (lie, manipulate others); engage in illegal activity (i.e., deal drugs, steal, prostitution); "isolate"; and continue to binge.

After reviewing the developed outcome, another group of program clients generally agreed that the scale reflected their lives on the better to worse ways of handling drug-use problems. Several people commented that the items that were near the bottom of list, or the worse ways of handling drug use, were not ways of handling the problem but were, in fact, the kinds of things that went on when your drug use was out of control. They described in graphic terms what this looked like:

To be focused every minute of every day on just getting the next bag of dope. My life is non-functional... I am a zombie. Wake up in the morning, get dressed and head straight for the corner to hustle up enough money to get that bag of dope.

Binging is like being on a mission. You go all the way out until everything is gone.... It can be one hour, a day or longer. It is when you have used all your resources and there is no more to be had. There is no one left for you to use or manipulate.

They also talked about why it was important to handle problems with drug use. One person admitted, "Your drug use is like a marriage, something you live with for life," and several clients talked about what their life looked like when they were able to handle their drug-use problems.

I need to have something with structure in my life to keep going... so you can function better... go forward... handle you apartment, raise your kids, keep yourself clean... stay out of jail and live a longer life. My everyday life is my life now.

The clients made several other insightful remarks about various items in this outcome. They commented on how "praying" helped them to function: "Praying helps me get things straight in my head." "It makes me strong and gives me more confidence,"; "Praying makes me more humble"; and "When I pray I feel more positive in my thinking." Regarding the item "follow a treatment plan," some people felt it was farther down on the scale because of the coercive aspect they associated with it: "Sometimes following a treatment plan is what you have to do because you have to see your parole officer every week, so you are forced to do it." In describing their experience in jail, many felt it did not help with problems with their drug use because it is very easy to get drugs in jail.

The clients did feel, however, that it was something to be avoided at all costs:

What you experience in jail makes you never want to go through that again.

It takes your freedom away. It changed me. Now I don't even steal a Hershey bar.

### Dealing with legal problems

The outcome 'dealing with legal problems' consists of a hierarchical scale of 11 items measuring the better to worse 'ways of handling legal problems'. The participants in the study considered better ways to handle legal problems were to pay, go see, and speak with a legal professional; address the problem yourself (go to law library, represent yourself, write to the judge); speak to a non-legal person (employer, counselor, parole officer, case manager); respect the law (by serving time, making court appearances); and learn from mistakes. The clients in the study considered worse ways of dealing with legal problems to be disrespecting the law (breaking the law, not respecting authority); facing the consequences of one's actions (serve time in prison or drug program, give up parental rights); avoiding legal responsibility (run from parole, leave the state, not show up in court, jump bail, ignore bench warrants); and relying on support from friends.

When a different group of program clients reviewed the outcome they agreed overall with the order of the items in the scale. They offered rich detail on specific items on the scale and insight into how drug users experience legal problems. Drug users confront a wide variety of legal problems, including being arrested for various drug-related charges; police harassment related to petty crimes like loitering or suspicion of a crime; legal problems related to one's children and the Bureau of Child Welfare; and taking part in a hearing to qualify for SSI.

Clients talked about their experience with legal professionals and items near the top of the scale. Many agreed that it was best when you could pay for an attorney, or, as one client put it, "Money talks and bullshit walks." However, they also realized that the steps in dealing with a professional first involved seeing and talking to one to find out the fee for services. Clients had varied experiences with professional attorneys, with several agreeing that legal aid lawyers were most helpful.

I prefer legal aid lawyers because they work from the heart and not by what you put in their pockets.

In housing I had a legal aid lawyer who helped me in a very positive way.

A private, paid lawyer to help me keep my kids did not do what he was supposed to do.

It was an SSI case and I had to pay and I got very little help or feedback from the lawyer at all.

As with other outcomes that have been reviewed, the participants in the study spoke favorably about trying to solve the problem themselves (item #4 on the scale).

It is good to do everything you can to help yourself first before you pay a legal professional or seek out their help.

You might be homeless, out there on the streets...and ready to come in and get your life together....

You need to investigate how to clean up any legal problems that may be lingering.

Sometimes friends may have gone through a similar experience and can tell you some of the steps they took to avoid jail or paying fines.

Clients talked about how they "learned from legal mistakes" (item #7) and what "disrespect the law" (item #8) meant to them. Learning from legal mistakes often involved experiencing the consequences when the police caught up with you:

I use to smoke my pipe out on the street and didn't care about the cops or anybody...When I saw the cops, I would run and hide and thought I got away from them. But when I came out of hiding, they were waiting for me and I got arrested.

Clients associated several different acts with "disrespecting the law" and often spoke of "testing" the authorities by jumping the subway turnstile, going to the bathroom in the street, jay-walking, and cursing out the cops.

Another interesting insight into the lives of drug users was how the clients felt that the law did not understand their ability to manage their drug use and lead a responsible life. Overall, they knew that when their drug use was out of control, they realized their respect for the law was "the farthest thing from your mind." One woman described how during a chaotic period she became suicidal and her children were taken away by the authorities. However, after she had effectively managed her drug use for some time and had sought out legal representation, even then she had not been allowed access to her children for the past six years.

## Discussion

The methodology used in the study contributed to the field of harm reduction and how to work more effectively with drug users. Meaningful outcomes for active drug users cannot be accurately measured in "either/or" terms (i.e., drug use vs. abstinence) or reflect a yardstick of achievement that is not culturally based in the lives of program clients. In that harm reduction programs work with drug users "where they are" and strive for incremental change that can be realistically accomplished, the results of the study represent the field's first attempt to establish relevant, culturally sensitive outcomes for measuring client and program success. Rather than using measures/standards developed by researchers in a different cultural world, the generated outcomes seek to more closely represent the lives of the population we are trying to understand and serve. The ethics and benefits of this participatory research/evaluation approach have been acknowledged by many [16-19].

This said, it should be noted that that the major limitation of the research was that it was done with a representative sample of only one harm reduction program in an urban area. And, although the sample included a variety of program clients whose drug use was characterized on a continuum from stable to chaotic, the external validity of the measures may be questionable for use with harm reduction programs having a different client population. The methodology is one that can be used by other harm reduction programs to help them identify ranked goals in each of the life areas for their particular population.

In addition to its methodological contribution, the study provided some important insights into drug users' lives and values, and an increased understanding of how this population envisions a better quality of life. Based on a number of items in the scaled outcomes, results showed that drug users often cited traditional measures in defining their life progress. Examples of this include having a legitimate job as a positive measure of source of income; being homeless and sleeping on the street as a measure of an undesirable housing situation; having an open and honest relationship with one's family as a measure of positive family relations; avoiding legal responsibility (i.e., jumping bail, running from parole) as a measure of an ineffective way of dealing with legal problems; and getting/staying clean of drugs as a positive measure of how to deal with drug use. It is generally felt that drug users' low ratings of certain activities as solutions to their problems (i.e., crime, prostitution) does not reflect a belief that these activities are inherently bad or immoral. Rather, these ratings reflect drug users' pragmatic nature and recognition that these activities can be impractical and dangerous.

Other findings from the qualitative research were somewhat counterintuitive to those outside the drug-use culture and reflect the realities of poverty, racism, social isolation, past trauma, and discrimination faced by individuals in this stigmatized population. For example, the study sample considered the very best way of making money was through entitlements (i.e., welfare, disability). This finding reflects the fact that since drug users struggle for day-to-day survival and often have a criminal record, many do not have the confidence, skills or opportunities to make a legitimate living in the dominant culture. It also reflects the reality that getting needed resources via entitlements is relatively easy and dependable for drug users while getting pay from a part-time job can be problematic when an employer in the drug trade can disappear on pay day. The stark realities of the drug user world were apparent in the fact that sex work and selling blood or body parts were ways that some individuals survived.

Findings related to the housing and nutrition outcomes also provided a window into the lives of many drug users. For example, being in jail was considered a more preferred housing arrangement than many forms of being homeless; eating out of the garbage – "dumpster diving" – was considered the worst way to try to get something good to eat. Interestingly, when it came to family relations, violence among family members was considered less problematic than being deceitful (stealing, gossip, lies) and may partially reflect the culture's shared value of openly expressing feelings. In regard to the outcome of connectedness to services and programs, it was not that surprising that the sample valued those services that addressed their day-to-day survival needs (entitlement benefits and programs related to housing, AIDS, and drug treatment), more than those that addressed less immediate needs (prevention, stress reduction, nutrition, and employment services).

Certain consistencies across the outcomes and items in the scales shed light on other values of drug users and the barriers they face. Overall, the sample indicated that the most preferred way of living was one in which they could try to work things out for themselves and remain independent of the dominant culture. This was seen in such examples as "having your own place to live"; "cooking food yourself as a way of getting good food"; "developing a better relationship with self as a way of improving yourself"; "praying and getting support from friends in order to deal with negative feelings"; "relying on home remedies to address health problems"; "admitting you have a problem and making amends as a way of handling problems with drug use"; and "going to a law library and doing research as a way of dealing with legal problems." These findings are not surprising, considering the fact that drug users often experience stigma and discrimination when they try to rely on the traditional service delivery system and, as a result, remain isolated and marginalized. It is also a finding that challenges the dominant view of drug users as lazy, dependent, and not wanting to change.

## Implications for the field

This preliminary research needs to be expanded in order to develop more valid and reliable outcome measures for the field of harm reduction. Other harm reduction programs are encouraged to conduct similar research with drug users in their locales and share their results. In addition, to validate that the outcomes are not simply research artifacts but reflective of the target population, field research can be conducted. For example, "hanging out" with the homeless and watching them as they spend the day looking for the best place to be homeless would help validate the outcome on housing. With measures that are science-based and evaluation results that can demonstrate the effectiveness of this approach to working with drug users, harm reduction programs will stand a better chance of receiving funding from potential donors.

In addition to their use, these measures for program outcome evaluation can also benefit the clinical component of programs. Case managers could collaboratively use the methodology in sessions with individual clients to develop relevant and realistic treatment plans. Future plans for behavior change developed in one-on-one sessions and using user-generated measures of progress have more potential for achievement than plans that do not consider how clients define progress.

## Conclusion

Clearly drug users have a set of workable solutions for meeting their own survival needs. Results of the present study show that they can often view institutions in the existing culture as irrelevant when addressing day-to-day living problems. The scales and rankings of many of the socially approved ways of solving life's problems show that just as there is an underground economy, there is a whole underground subculture where those marginalized from the mainstream have developed a culture with its own set of relevant structures, informal relationships, and home-grown recipes for addressing life's challenges.

These study findings convey an understanding of drug users as people who are interested in positive change in all areas of their lives, and that by empowering them in a process to identify their own goals, they may be more motivated and engaged in the program. The findings also provide some initial empirical evidence that challenges traditional ways of measuring drug use based solely on quantity and frequency. Results suggest that more appropriate measures may be the extent to which the drug users organize their lives around drug use, the extent to which drug use is integrated into their lives, and the extent to which drug use negatively impacts other aspects of their lives. They also suggest that harm reduction and other programs serving active drug users and other marginalized people should not rely on institutionalized, provider-defined solutions to the problems in living faced by their clients. Rather, drug users should be assisted with problem solving by being encouraged to consult their own set of culturally shared solutions.

## Competing interests

None declared.

## Authors' Contributions

T.R. conceived the study, designed the research, and provided input on the manuscript. S.R. coordinated the study, provided oversight on data collection, analyzed the data and wrote the manuscript. Both authors read and approved the final manuscript.

## Supplementary Material

Additional file 1 Outcomes of Harm Reduction Programming to Measure Incremental Change from Better to Worse. This is a tabular form of all categories of behavior discussed in the paper. Click here for file
