# Supplementary material for: How do drug users define their progress in harm reduction programs? Qualitative research to develop user-generated outcomes
Source: Harm Reduct J. 2004 Aug 26;1:8. doi: 10.1186/1477-7517-1-8 (PMC516446; doi:10.1186/1477-7517-1-8)
Supplement: Additional file 1 — Outcomes of Harm Reduction Programming to Measure Incremental Change from Better to Worse. This is a tabular form of all categories of behavior discussed in the paper. [file 1477-7517-1-8-S1.doc]

**Table 1: Outcomes of Harm Reduction Programming to Measure Incremental Change from Better to Worse**

| Ways of Making Money 1. Entitlements (welfare,  disability)  2. Job (employment, peddling,  odd jobs, volunteer)  3. Family money, gifts  4. Borrowing  _ _ _ _ _ _ _ _ _ _ _ _ _ _ _ _  5. Hustling, police informant  6. Stealing (boosting,  embezzle)  7. Drug trade (selling,  holding,  transporting)  8. Pan handling, collecting  cans  9. More serious criminal acts  (robbery, loan shark, hit man)  10. Sex work  11. Selling blood, body organs | **Places to Live**  1. House you rent or own 2. Friend’s home  3. Apt/room you rent or own 4. Drug program  5. Family member’s home  6. Housing with social program   1. Institutionalized housing   (shelter, hospital, hotel)   1. Living on street/   subway/bus station  _ _ _ _ _ _ _ _ _ _ _ _ _ _ _ _  9. Jail  10. Sleeping in cars/tent/  abandoned building  11. Sleeping in tunnels/roof/  parks/stairways | Ways/Places to Get Something Good to Eat  1. Cook food yourself 2. Food from friends/family  3. Food from market 4. Free food 5. Buy food (foodstamps/   money)  6. Go out to restaurant  _ _ _ _ _ _ _ _ _ _ _ _ _ _ _ _  7. Beg for food 8. Steal food 9. Food from facilities (jail,  hospital)  10. Provide your own food  (hunt, fish)  11. Food from garbage | Types of Services/ Programs   1. Housing 2. AIDS related 3. Mental health 4. Drug treatment 5. Entitlements 6. Harm reduction   _ _ _ _ _ _ _ _ _ _ _ _ _ _ _ _   1. Mainstream institutions   (churches, library, legal)   1. Get connected services   (transport, escort)   1. Support services (AA, NA,   women’s group, friends)   1. Prevention services (parenting, domestic violence)   11. Stress reduction  12. Work-related (WEP) | **Ways to Handle Legal Problems**  1. Pay for a legal professional  2. Go see a legal professional  3. Speak with a legal professional  4. Address the problem yourself (do  research, write to judge)  5. Speak to non-legal person  (counselor, case manager)  6. Respect the law (serve time in central booking, make court appearances)  7. Learn from legal mistakes  _ _ _ _ _ _ _ _ _ _ _ _ _ _ _ _ _  8. Disrespect the law, authorities  9. Face consequences (give up  parental rights, go to appeal)  10. Avoid legal responsibility (jump  bail, don’t pay fines)  11. Get help from friends | Better **Worse** |
| --- | --- | --- | --- | --- | --- |
| Types of Family Relations 1. Love for family 2. Special family gatherings 3. Positive communication  (open, honest, patient) 4. Interactive activities  (picnics, play games)  5. Argue  6. Support, respect 7. Spend quality time  together 8. Passive activities (TV,  movies, music)  _ _ _ _ _ _ _ _ _ _ _ _ _ _ _ _  9. Lack of respect  10. Negative attitudes  (jealous, judgmental) 11. Conflicting lifestyles  between members family members 12. Abusive relations  (physical, sexual) 13. Difficult financial relations 14. .Abandonment of family  15. Deceitful relations (lying,  stealing, gossip) | Ways of Improving Yourself 1. Developing more self respect  2. Relating better to others  3. Getting/ staying clean  4. Becoming more spiritual   1. Taking part in self-help 2. groups   6. Working/ developing work  skills  7. Reducing stress (meditation,  yoga)  _ _ _ _ _ _ _ _ _ _ __ _ _ _ _  8. Helping others (get a job,  babysit for children)  9. Caring for self (go to dentist,  taking medications, diet)  10. Being more responsible (live  on a budget, accomplish  goals)  11. Behaving myself (staying out of trouble, stop lying)  12. Taking up hobbies (artwork,  fishing, hunting) | **Ways to Handle Negative Feelings**  1. Get support (support groups,  friends)  2. Spiritual help (pray, church)  3. Professional help (case  manager, counselor, doctor)  4. Work a job or volunteer  5. Diversions (ball game, beach,  singing)  6. Stress reduction (meditation,  smoking)  _ _ _ _ __ _ _ _ _ _ _ _ _ _ _  7. Physical activities (sports,  cooking)  8. Self abuse (anorexia, suicide)  9. Abuse of others  10. Social relationships (visit  person in jail, get married)  11. Withdrawal, isolate  12. Illegal activities (drugs,  gamble) | **Ways to Handle Health Problems**  1. Home remedies (cleansing,  praying)  2. Stress reduction (positive  affirmations, meditation)  3. Drug treatment/ therapy  4. Clean living (reduce drug use,  take meds, stop smoking)  5. See doctor  6. Health screening (check for  diabetes, STDs, etc.)  _ _ _ _ _ _ _ _ _ _ _ _ _ _ _ _ _  7. Nutritional diet  8. Educate yourself about health  9. Exercise  10. Alternative therapies (psychic,  herbs, fasting)  11. Negative emotions (denial,  anger, depression, suicide)  12. Use illegal drugs | **Ways to Handle Problems with Drug Use**  1. Admit the problem  2. Pray  3. Get social support  4. Go into treatment  5. Quit using  6. Get help from therapist  7. Stay distracted  8. Avoid drug culture  _ _ _ _ _ _ _ _ _ _ _ _ _ _ _ _ _  9. Follow treatment plan  10. Get family support  11. Get spiritual guidance (NA,AA,  minister)  12. Jail  13. Reflect on pain associated with  drug use  14. Be deceitful (lie, manipulate)  15. Take part in illegal activity  16. Isolate  17. Use drugs, binge | **Better**  **Worse** |
